# Combination of Electrochemotherapy with Radiotherapy: A Comprehensive, Systematic, PRISMA-Compliant Review of Efficacy and Potential Radiosensitizing Effects in Tumor Control

**DOI:** 10.3390/curroncol30110719

**Published:** 2023-11-13

**Authors:** Martina Ferioli, Anna M. Perrone, Milly Buwenge, Alessandra Arcelli, Maria Vadala’, Bruno Fionda, Maria C. Malato, Pierandrea De Iaco, Claudio Zamagni, Silvia Cammelli, Luca Tagliaferri, Alessio G. Morganti

**Affiliations:** 1 Radiation Oncology, Department of Medical and Surgical Sciences (DIMEC), University of Bologna, 40138 Bologna, Italy; myriam.perrone@aosp.bo.it (A.M.P.); milly.buwenge2@unibo.it (M.B.); alessandra.arcelli@aosp.bo.it (A.A.); mariachiara.malato@studio.unibo.it (M.C.M.); pierandrea.deiaco@unibo.it (P.D.I.); silvia.cammelli2@unibo.it (S.C.); alessio.morganti2@unibo.it (A.G.M.); 2 Division of Oncologic Gynaecology, IRCCS Azienda Ospedaliero-Universitaria di Bologna, 40138 Bologna, Italy; 3 Radiation Oncology, IRCCS Azienda Ospedaliero-Universitaria di Bologna, 40138 Bologna, Italy; 4 Nuclear Medicine, IRCCS Azienda Ospedaliero-Universitaria di Bologna, 40138 Bologna, Italy; maria.vadala@aosp.bo.it; 5 Dipartimento di Diagnostica per Immagini, Radioterapia Oncologica ed Ematologia, Fondazione Policlinico Universitario “A. Gemelli” IRCCS, UOC di Radioterapia Oncologica, 00168 Roma, Italy; bruno.fionda@policlinicogemelli.it (B.F.); luca.tagliaferri@policlinicogemelli.it (L.T.); 6 Oncologia Medica Addarii, IRCCS Azienda Ospedaliero-Universitaria di Bologna, 40138 Bologna, Italy; claudio.zamagni@aosp.bo.it

**Keywords:** electrochemotherapy, electroporation, radiotherapy, radiosensitivity, systematic review

## Abstract

Radiotherapy (RT) and electrochemotherapy (ECT) are established local treatments for cancer. While effective, both therapies have limitations, especially in treating bulky and poorly oxygenated tumors. ECT has emerged as a promising palliative treatment, raising interest in exploring its combination with RT to enhance tumor response. However, the potential benefits and challenges of combining these treatments remain unclear. A systematic review was conducted following PRISMA guidelines. PubMed, Scopus, and Cochrane libraries were searched. Studies were screened and selected based on predefined inclusion and exclusion criteria. Ten studies were included, comprising in vitro and in vivo experiments. Different tumor types were treated with ECT alone or in combination with RT. ECT plus RT demonstrated superior tumor response compared to that under single therapies or other combinations, regardless of the cytotoxic agent and RT dose. However, no study demonstrated a clear superadditive effect in cell survival curves, suggesting inconclusive evidence of specific ECT-induced radiosensitization. Toxicity data were limited. In conclusion, the combination of ECT and RT consistently improved tumor response compared to that with individual therapies, supporting the potential benefit of their combination. However, evidence for a specific ECT-induced radiosensitization effect is currently lacking. Additional investigations are necessary to elucidate the potential benefits of this combination therapy.

## 1. Introduction

Radiotherapy (RT) is a cornerstone in cancer treatment, with applications in both curative and palliative settings depending on the clinical context and patient’s condition. RT effectively controls local tumors as a single treatment modality, enhances local control before or after surgery in operated patients, and alleviates tumor-induced symptoms. However, its efficacy may be limited, particularly in bulky and poorly oxygenated tumors, due to the constraints of administering a dose that avoids severe side effects to surrounding healthy organs [1,2].

Electrochemotherapy (ECT) is a relatively recent addition to cancer treatment, primarily used in the palliative setting. ECT involves the synergistic application of electrical pulses and chemotherapy, where specialized electrodes are directly inserted into and around the tumor. Short electrical pulses are then delivered, creating transient pores in the tumor cell membranes (electroporation, EP), enabling the increased penetration of chemotherapeutic drugs into tumor cells, thereby improving their effectiveness. The drug used (usually Bleomycin or Cisplatin) can be administered intravenously or intratumorally, and after 8 min, electric pulses can be delivered to tumor area with a safe margin. ECT’s unique benefit lies in its selectivity for cancer cells, sparing healthy surrounding tissues; cell death occurs via apoptosis due to double-strand breaks caused by the chemotherapeutic drug. There is a selective killing of the dividing tumor cell that replicates rapidly and synchronously, leading to necrosis, as opposed to surrounding healthy tissue. Encouraging outcomes from this procedure demonstrate its potential to improve tumor control and overall patient outcomes, thus solidifying its position as a valuable component in the array of cancer treatment modalities [3,4,5].

Given the aforementioned limitations of RT, various researchers have explored the prospect of combining RT with ECT in experimental models to enhance local tumor control [6]. Some studies showed that ECT exhibits a radiosensitizing effect, potentially improving the antitumor impact of RT [7]. However, the available evidence is currently dispersed and inconsistent in terms of experimental design and model characteristics.

Therefore, the primary aim of this review is to comprehensively synthesize the existing literature on the radiosensitizing effect of RT in combination with ECT, seeking to provide a consolidated and coherent understanding of this intriguing therapeutic approach.

## 2. Materials and Methods

### 2.1. Study Protocol Registration and Guidelines

Prior to commencing literature screening, we registered the protocol for this systematic review in the PROSPERO international register on 8 October 2019 (registration number CRD42020153811) [8]. The PRISMA (Preferred Reporting Items for Systematic Reviews and Meta-Analyses) guidelines were followed to ensure comprehensive and transparent analysis of the data [9]. The primary endpoints assessed in this review included in vitro and in vivo tumor control, as well as toxicity after EP/ECT combined with ionizing radiation (IR).

### 2.2. Bibliographic Search

The literature search encompassed three major databases, PubMed, Scopus, and the Cochrane libraries, without time limitations. This the search strategy in PubMed database (accessed on 18 october 2020) (accessed on: ((((radiosensitization) OR Radio-sensitization)) OR radiosensitivity) AND ((((electroporation) OR electrochemotherapy)) AND ((radiotherapy) OR radiation therapy)); Scopus database: (TITLE-ABS-KEY ((radiosensitization OR radiosensitivity)) OR TITLE-ABS-KEY (radio-sensitization) AND TITLE-ABS-KEY (electroporation OR electrochemotherapy) AND TITLE-ABS-KEY (radiotherapy) OR TITLE-ABS-KEY (radiation AND therapy)); Cochrane Library: “radiosensitivity” in Title Abstract Keyword OR “radiosensitisation” in Title Abstract Keyword OR “radio sensitivity” in Title Abstract Keyword AND “electroporation” in Title Abstract Keyword OR “electrochemotherapy” in Title Abstract Keyword. In addition to the electronic search, further relevant papers were identified from the reference lists of selected publications.

### 2.3. Inclusion Criteria

This systematic review encompassed studies published in English, including both preclinical (in vitro or in vivo) and clinical (retrospective- or prospective-design) investigations. Excluded from this review were papers not reporting tumor control, studies involving EP with the cell transfection of macromolecules unrelated to anticancer drugs, papers involving EP or ECT applied to non-cancerous conditions, studies lacking detailed data on each treatment type or combination, systematic or narrative reviews, meta-analyses, guidelines or recommendations, case reports, letter-commentaries-editorials, imaging studies, surveys, and reports containing duplicate data.

### 2.4. Study Selection

Two authors (MF, AMP) independently screened papers at the title and abstract level, excluding duplicate entries. In the event of any disagreements during this process, a final decision was reached through a discussion with the senior author (AGM). The full text of all potentially relevant articles was independently examined by the same authors, and any discrepancies were resolved as previously described.

### 2.5. Data Extraction

Data from the selected papers were independently collected by two authors (MB and AA) using a predetermined form, which included information on study design, tumor histology, characteristics of ECT/EP and RT, tumor control outcomes, toxicity assessments, and radiosensitivity parameters. Following data collection, the final results were cross-validated by the senior author to address any discrepancies. In case of conflicting data, thorough discussions were conducted to arrive at a consensus.

### 2.6. Quality Assessment

A quality assessment of the included studies was conducted based on the clarity of the description regarding (i) the cell lines, animal models, or patient populations used; (ii) the treatment characteristics and timeline employed; and (iii) the reporting of tumor response and local control data. Additionally, the extent of missing data in each study was recorded for further evaluation.

## 3. Results

### 3.1. Search Results

**Figure 1:**
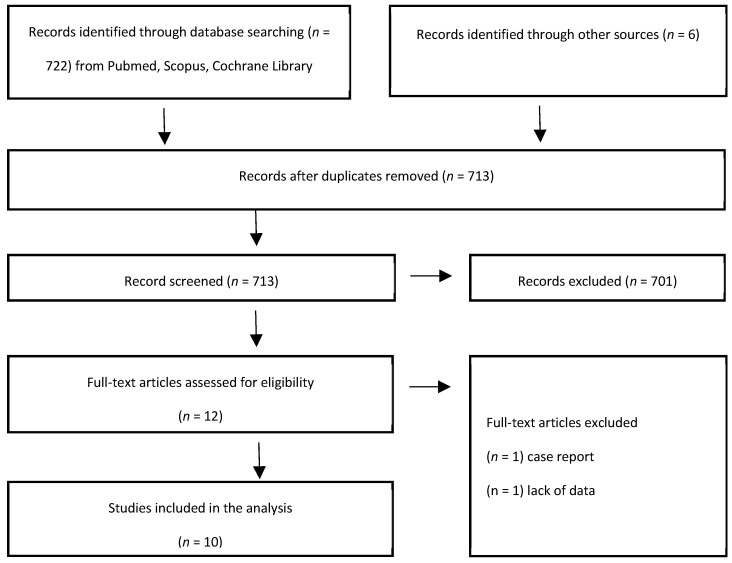
Flow chart study selection diagram.

The PRISMA flowchart (Figure 1) presents the process of study selection. Initially, 722 studies in total were identified from literature databases, and an additional six records were found from other sources, resulting in a total of 713 papers after removing duplicates. After screening at the title and abstract level, 12 full texts were assessed. Ultimately, 10 studies were included in the analysis [10,11,12,13,14,15,16,17,18,19], while 1 paper was excluded due to it being a case report and another was excluded due to a lack of data and separate clinical outcomes for each treatment combination.

### 3.2. Characteristics of Included Studies

All included studies reported preclinical data, with two studies performed in vitro [13,19], five studies conducted in vivo [11,12,16,17,18], and three studies encompassing both in vitro and in vivo investigations [8,12,13]. The detailed characteristics of the included studies are presented in Table 1 (in vitro) and Table 2 (in vivo).

### 3.3. Tumor and Treatment Characteristics

ECT/EP and IR treatment characteristics are summarized in Table 3 (in vitro) and Table 4 (in vivo). 

The analyzed studies covered tumors with various histological types, predominantly sarcomas [10,11,14,15,16] and carcinomas [12,13,16,17,18]. In vitro studies utilized human [13] or murine tumor cells [10,14,15,19], while preclinical in vivo studies utilized murine models [10,11,12,14,15,16,17,19,20].

Among the included studies, eight treated tumors with ECT, and two treated tumors with EP alone [10,13]. ECT was performed using different anticancer drugs, including cisplatin (CDDP) in four studies [12,15,18,19], bleomycin (BLM) in two studies [14,16], doxorubicin (DOX) in one study [11], and tirapazamine (TPZ) in the last study [17]. In vivo, the drug was administered intravenously by four authors [14,15,16,18] and intratumorally by two authors [11,12]. Finally, in Maxim’s et al. study [17], ECT was based on intraperitoneal TPZ infusion.

Regarding pulse application, five studies applied one train of eight pulses [14,15,17,18,19], two studies employed ten trains of eight pulses [11,12], two studies used two trains of four pulses [12,16], and one study applied a single pulse [13]. The electric field intensity ranged between 1000 and 1300 V/cm, the duration of pulses varied from 100 to 200 µs, and the frequency was 1 Hz in all studies, except for one report that did not provide these data [13].

In terms of IR, all studies reported the total dose, dose per fraction, dose rate, IR beam type, and voltage. Nine authors delivered the dose in a single fraction [10,11,12,13,14,15,17,18,19], while Kranjc et al. [16] used a hypofractionated IR with one to five fractions. The dose per fraction ranged between 2 and 50 Gy, with better tumor control and a greater immunogenic effect with a higher dose per fraction and a fractionated schedule. In four, a single IR dose level was delivered [10,11,17,18], while in five studies the IR dose was escalated [13,14,15,16,19]. One study used two different dose levels and compared the tumor response between the two groups [12].

### 3.4. Tumor Control and Toxicity in In Vitro Studies

Among the in vitro studies (Table 5), two papers reported the drug concentration required to reduce cell survival by 50% (IC50) [15,19], and one reported the IR dose needed to achieve 50% of cell survival (LD50) [13].

In vitro studies consistently demonstrated that the combination of ECT plus IR required lower drug concentrations to reduce cell survival compared to those required in other treatment combinations [15,19]. The most significant differences were observed when comparing chemotherapy (CHT) alone versus ECT plus IR in terms of IC50 (120 vs. 2 µg/mL). Regardless of the cytotoxic agent type and tumor/cell histological type, the combination of ECT plus IR consistently outperformed other treatment combinations, reaffirming the significant impact of EP in improving tumor control.

Shil et al. [10] and Yadollahpour et al. [13] investigated EP plus IR. The former study showed a two-fold increase in intracellular reactive oxygen species (ROS) generation, a significant decrease in cellular viability, and changes in membrane fluidity. The latter reported a sharper reduction in the cell survival fraction (*p* < 0.05) with EP plus IR compared to that under EP alone, indicating increased sensitivity to IR by a factor of 1.36.

### 3.5. Tumor Response and Toxicity in In Vivo Studies

Tumor response was assessed using different outcomes (Table 6), including TCD50 [14,15], TDT [11,12,16,18], TGD [11,12,16,17,18], and the percentage reduction in tumor volume [10].

In vivo studies consistently showed superior results with the combination of ECT plus IR. This combination reduced TCD50 [14,15] and improved TGD [11,12,16,17,18] compared to those under single treatments or other treatment combinations, irrespective of the cytotoxic agent and IR dose. The combination of ECT plus IR resulted in lower TCD50 compared to that under the combination of EP plus IR (14.2 vs. 23.5 Gy) and IR alone (12.4 vs. 23.1 Gy) [14,15]. Moreover, ECT plus IR exhibited the longest TDT, especially when compared to that under EP alone (44.5 vs. 4.5 days; 30.3 vs. 6.6 days). Additionally, tumor growth delay (TGD) was significantly prolonged with single-fraction IR compared to multifractionated IR [16], and also with a higher dose per fraction (5 vs. 3 Gy). ECT plus IR demonstrated superior efficacy compared to that of CHT alone (17.5 vs. 1.0 days; 1.62 vs. 0.66 days; 38.0 vs. 1.1 days) [11,16,17], EP alone (40.6 vs. 0.6 days; 25.7 vs. 2.0 days) [12,18], and EP plus IR (38.6 vs. 8.8 days) [12]. Additionally, Kranjc et al. reported a greater impact on TGD with single-dose IR [16]. Lastly, Shil et al. [10] reported that the average tumor volume after ECT plus IR was 51% of that in the control group.

### 3.6. Quality Assessment

All studies provided a clear definition of cell lines, animal models, or patient populations, as well as treatment characteristics. However, tumor response was reported differently among the papers, with variations in the subgroups of comparison. Moreover, two studies did not define the timing of treatment combinations [10,13], and toxicity data were reported in only three out of eight studies [12,14,16].

## 4. Discussion

Electrochemotherapy (ECT) and radiotherapy (RT) are well-established local treatments for tumors, and both have demonstrated efficacy. However, their effectiveness may be limited, particularly for bulky tumors. To enhance the efficacy of RT, combinations of various systemic therapies, including chemotherapy, hormonal therapy, targeted treatments, and immunotherapy, have been explored. Additionally, the combination of RT with other local treatments such as hyperthermia [20], hypothermia [21], and high-intensity focused ultrasound (HIFU) [22] has shown promise in improving treatment outcomes. The increasing body of evidence supporting the efficacy of ECT in tumor control has prompted interest in combining RT and ECT to potentially amplify tumor response. 

Nevertheless, the inherent benefits of combining different local treatments are not always apparent. Prior to introducing a combination of two local treatments for cancer into clinical practice, careful consideration must be given to the risk of overlapping toxicity and potential protective effects on the tumor. For instance, in some experimental models, hypothermia demonstrated an increase in radiosensibility [23] while in others, it exhibited a radioprotective effect [21]. 

From a theoretical perspective, the possibility of radiation protection induced via ECT cannot be disregarded, especially if ECT is administered before RT. In fact, ECT is known to produce a “vascular lock”, which may lead to increased hypoxia and reduced radiosensitivity. However, the results of our analysis do not support this hypothesis, as no study demonstrated a reduction in tumor response when ECT and RT were combined.

Once we exclude a radioprotective effect, several alternatives become plausible. One possibility is that two treatments independently induce cell killing, resulting in an improved tumor response compared to that under single therapies, without significant interaction between treatments. Based on our review, this effect seems to be confirmed by all published studies, regardless of the experimental model. Indeed, all studies included in our review exhibited an improved tumor response in experiments involving the ECT-RT combination. Moreover, EP alone has a radiosensistizing effect because it induces immunogenic cell death through the liberation of mediators named damage-associated molecular patterns (DAMPs) that, together with tumor antigens, activate antigen presenting dendritic cells [24]. These molecules are also released after CHT with CDDP, leading to a potential synergistic effect in ECT treatment. One of these DAMPs, called calreticulin, is exposed after EP, promoting the subsequent tumor infiltration by granzyme B-cells, However, its release was not enhanced only via exposure to a chemotherapeutic drug [25].

However, whether or not we can categorize this effect as ECT-induced radiosensitization remains to be determined. In other words, it is yet to be proven whether or not ECT can genuinely increase the radiosensitivity of tumor cells and tissues, leading to an “enhancement effect” or “super-additive effect”, rather than a simple additive effect resulting from independent cell killing. None of the analyzed studies demonstrated a superadditive effect from the ECT-RT combination, which would require demonstrating an increase in the slope of the cell survival curve. Moreover, no studies have shown the specific efficacy of either therapy in cell subpopulations resistant to the other therapy.

Furthermore, there has been a lack of investigation into whether or not the ECT-RT combination results in increased toxicity to healthy organs. Evaluating this aspect would be crucial in determining whether or not the combined treatment truly enhances the therapeutic ratio, achieving a greater response without increasing toxicity. As such, we do not find it justified to claim that ECT has a radiosensitizing effect, contrary to one previous systematic review in the literature [6] and four publications included in our review [14,15,16,19], which assert the existence of this effect.

However, our analysis has some limitations. The response to treatment was reported using different parameters among both in vitro and in vivo studies. Moreover, the subgroups of treatment combinations were not the same among studies, making it difficult to make any comparison between them. Regarding ECT treatment, different drugs and different routes of administration were used. Similarly, the dose and dose rate of radiation were heterogeneous, and only Kranjc et al. tested a fractionated dose [16]. Finally, toxicity was reported only in three studies [12,14,16].

Therefore, further studies are required to ascertain the radiosensitizing effect of ECT. These studies should aim to (i) evaluate the toxic effects on healthy organs when combining ECT and RT with different timings and dosages; (ii) assess whether or not the addition of ECT modifies the slope of the survival curve of irradiated cells or merely displaces it, as observed in a purely “additive” effect; and (iii) investigate whether or not both treatments exhibit specific efficacy in cellular subpopulations that are more resistant to the other therapy.

## 5. Conclusions

Traditionally, radiosensitizers [26], or “pure radiosensitizers” [27] have been defined as compounds or drugs without inherent antitumor activity but with the capacity to increase tumor radiosensitivity, as exemplified by nitroimidazoles [28]. Moreover, a radiosensitizer could act by increasing the degree of DNA damage, as cytotoxic drug delivered into cells during ECT, or by disturbing the cell cycle [29]. Consequently, due to its specific antitumor activity, ECT cannot be classified as a pure radiosensitizer. Conversely, “ideal radiosensitizers” refer to treatments that, when combined with RT, enhance the tumor response without increasing radio-induced toxicity to healthy tissues [30]. However, no study has demonstrated such an improvement in the therapeutic ratio following the combination of ECT with RT. Therefore, based on the current state of knowledge, ECT cannot be defined as either a pure radiosensitizer or an ideal radiosensitizer. Further research is warranted to elucidate its role in enhancing the effect of RT on tumors.

## Figures and Tables

**Table 1:** Study characteristics (in vitro series).

Author/Year	Tumor Cells
Kranjc S, 2003 [15]	LPB murine sarcoma cells
Kranjc S, 2003 [15]	SCK murine mammary carcinoma cells and EAT-E cells
Kranjc S, 2005 [14]	LPB murine sarcoma cells and tumors
Shil P, 2005 [10]	Murine fibrosarcoma
Yadollahpour A, 2018 [13]	Human colorectal cancer cell line HT-29

**Table 2:** Study characteristics (in vivo series).

Author/Year	Study Type	Tumor Cells Histology
Sersa G, 2000 [18]	Preclinical	Ehrlich-Lettre ascites carcinoma in CBA mice
Kranjc S, 2003 [15]	Preclinical	LPB murine sarcoma
Maxim P.G, 2004 [17]	Preclinical	Squamous cell carcinoma in C3H mice
Kranjc S, 2005 [14]	Preclinical	LPB murine sarcoma
Shil P, 2005 [10]	Preclinical	Murine fibrosarcoma
Shil P, 2006 [11]	Preclinical	Murine fibrosarcoma
Kranjc S, 2009 [16]	Preclinical	Sarcoma SA-1 and mammary adenocarcinoma CaNT in CBA and A/J mice
Raeisi E, 2012 [12]	Preclinical	Invasive ductal carcinoma tumors in Balb/C mice

**Table 3:** Treatment characteristics (in vitro series).

	ECT/EP Treatment					IR Treatment				
Author/Year	Drug	n° Pulses	Intensity (V/cm)	Duration (µs)	Frequency (Hz)	Tot Dose (Gy)	Dose/Fract (Gy)	Rad Type	Dose Rate (Gy/Min)	Kilo/Mega Voltage
Kranjc S, 2003 [15]	CDDP	8	1200	100	1	2–8	2–8	X-ray	2	220 kV
Kranjc S, 2003 [15]	CDDP	8	1000	100	1	2–8	2–8	X-ray	2	220 kV
Kranjc S, 2005 [14]	BLM	8	1200	100	1	2–8	2–8	X-ray	2	220 kV
Shil P, 2005 [10]	NONE	8 × 10	1000	200	1	2	2	Co60 γ-rays	0.37	1.25 MV
Yadollahpour A, 2018 [13]	NONE	1	1200	100	NR	0–8	0–8	X-ray	3	6 MV

µs: microseconds; BLM: bleomycin; CDDP: cis-Diamminedichloroplatinum; Co60: Cobalt-60; ECT: electrochemotherapy; EP: electroporation; Gy: gray; Hz: hertz; IR: irradiation; kV: kilovoltage; MV: megavoltage; V/cm: volt per centimeter.

**Table 4:** Treatment characteristics (in vivo series).

	ECT/EP Treatment						IR Treatment				
Author/Year	Drug	RoA	n° Pulses	Intensity (V/cm)	Duration (µs)	Frequency (Hz)	Total Dose (Gy)	Dose/Fraction (Gy)	Radiation Type	Dose Rate (Gy/Min)	Kilo/Mega Voltage
Sersa G, 2000 [18]	CDDP	IV	8	1300	100	1	15	15	X-ray	2.1	220 kV
Kranjc S, 2003 [15]	CDDP	IV	8	1300	100	1	5–50	5–50	X-ray	2.1	220 kV
Maxim P.G, 2004 [17]	TPZ	IP	8	1200	100	1	7	7	X-ray	0.83	250 kV
Kranjc S, 2005 [14]	BLM	IV	8	1200	100	1	5–50	5–50	X-ray	2.1	220 kV
Shil P, 2005 [10]	NONE	NA	8 × 10	1000	200	1	2	2	Co60 γ-rays	0.37	1.25 MV
Shil P, 2006 [11]	DOX	IT	8 × 10	1000	200	1	2	2	Co60 γ-rays	0.37	1.25 MV
Kranjc S, 2009 [16]	BLM	IV	4 × 2	1300	100	1	10–20	2–20	X-ray	2.2	220 kV
Raeisi E, 2012 [12]	CDDP	IT	4 × 2	1000	100	1	3\5	3\5	Co60 γ-rays	0.6 (3 Gy)–0.71 (5 Gy)	1.25 MV

µs: microseconds; BLM: bleomycin; CDDP: cis-Diamminedichloroplatinum; Co60: Cobalt-60; DOX: doxorubicine; ECT: electrochemotherapy; EP: electroporation; Gy: gray; Hz: hertz; IP: intraperitoneal; IR: irradiation; IT: intratumoral; IV: intravenous; NA: not applicable; RoA: road of administration; TPZ: tirapazamine; V/cm: volt per centimeter.

**Table 5:** Tumor control (in vitro).

Author/Year	Treatment Groups	IC50 (µg/mL)		LD50 (Gy)	Cell Viability	EF
Kranjc S., 2003 [15]	CDDP	120		-	-	-
	CDDP + EP	4		-	-	-
	CDDP + IR	23		-	-	-
	**CDDP + EP + IR**	**2**		-	-	-
	IR	-		-	-	-
	EP + IR	-		-	-	-
	CONTROLS	-		-	-	-
	EP	-		-	-	-
Kranjc S., 2003 [15]	CDDP	SCK 14.8	EAT-E 48.5	-	-	-
	CDDP + EP	3.4	2.2	-	-	-
	CDDP + IR	8.0	22	-	-	-
	**CDDP + EP + IR**	**0.9**	**0.9**	-	-	-
Kranjc S., 2005 [14]	CONTROLS	-		-	-	-
	IR	-		-	-	-
	BLM + IR	-		-	-	1.19
	EP + IR	-		-	-	1.25
	**BLM + EP + IR**	**-**		**-**	**-**	**1.53**
Shil P., 2005 [10]		-		-	5% reduction	-
Yadollahpour A., 2018 [13]	IR	-		3.97	-	-
	**EP + IR**	**-**		**2.9**	-	-

BLM: bleomycin; CDDP: cis-Diamminedichloroplatinum; EAT-E: Ehrlich ascites carcinoma cells; EF: enhancement factor; EP: electroporation; Gy: gray; IC50: drug concentration required to reduce cell survival for 50%; IR: irradiation. (The parts in bold highlight the association of treatments).

**Table 6:** Tumor control (in vivo).

Author/Year	Treatment Groups	TCD50 (Gy)	DT (Days)		TGD (Days)		OR (%)	EF	Decreased Tumor Volume vs. Controls (%)	Toxicity
Sersa G., 2000 [18]	CONTROLS	-	3.9		Nr		-	-	-	-
	CDDP	-	5.4		1.5		-	-	-	-
	EP	-	4.5		0.6		-	-	-	-
	IR	-	17.4		13.5		-	-	-	-
	CDDP + EP	-	13.4		9.5		-	-	-	-
	CDDP + IR	-	19.5		15.6		-	-	-	-
	**CDDP + EP + IR**	**-**	**44.5**		**40.6**		-	-	-	-
	EP + IR	-	24.2		20.3		-	-	-	-
Kranjc S., 2003 [15]	CDDP	-	-		-		-	-	-	-
	CDDP + EP	-	-		-		-	-	-	-
	CDDP + IR	19.6	-		-		-	1.1	-	-
	**CDDP + EP + IR**	**14.2**	**-**		-		-	1.6	-	-
	IR	22.1	-		-		-	-	-	-
	EP + IR	23.5	-		-		-	0.9	-	-
	CONTROLS	-	-		-		-	-	-	-
	EP	-	-		-		-	-	-	-
Maxim P.G., 2004 [17]	CONTROLS	-	-		large tumors	small tumors	-	-	-	-
	TPZ	-	-		1.0	2.0	-	-	-	-
	TPZ + EP	-	-		7.5	7.5	-	-	-	-
	TPZ + IR	-	-		10.5	7.0	-	-	-	-
	**TPZ + EP + IR**	**-**	-		**17.5**	**13.0**	-	-	-	-
	EP + IR	-	-		3.5	3.0	-	-	-	-
Kranjc S., 2005 [14]	CONTROLS	-	-	-	-	-	-	-	-	hair loss in the irradiated area
	IR	23.1	-	-	-	-	-	-	-	
	BLM + IR	22.8	-	-	-	-	-	1.0	-	
	EP + IR	22.1	-	-	-	-	-	1.0	-	
	**BLM + EP + IR**	**12.4**	-	-	-	-	-	1.9	-	
Shil P., 2005 [10]	CONTROLS	-	-		-		-	-	-	-
	EP	-	-		-		-	-	-	-
	IR	-	-		-		-	-	-	-
	**EP + IR**	-	-		-		-	-	**51**	-
Shil P., 2006 [11]	CONTROLS	-	1.28		nr		-	-	-	-
	VEHICLE CONTROL	-	1.30		nr		-	-	-	-
	EP	-	2		0.72		-	-	85	-
	IR	-	1.82		0.54		-	-	82	-
	DOX	-	1.94		0.66		-	-	88	-
	DOX + EP	-	2.5		1.22		-	-	57	-
	IR + EP	-	2.78		1.5		-	-	-	-
	DOX + IR	-	2.48		1.2		-	-	52.5	-
	**IR + DOX + EP**	**-**	**3**		**1.72**		-	-	49	-
Kranjc S., 2009 [16]	CONTROLS	-	(SA-1) 2.1	(CaNT) 2.2	(SA-1)	(CaNT)	-	-	-	IR (SD vs. FD): more toxicity on normal skin and more body weight loss
	EP	-	3.8	3.6	1.7	1.4	-	-	-	
	BLM	-	3.2	2.6	1.1	0.4	-	-	-	
	BLM + EP	-	22.3	17.8	20.2	15.6	-	-	-	
	IR (SD)	-	15	17.1	12.9	14.9	-	-	-	
	BLM + IR (SD)	-	15.1	17.7	13.0	15.5	-	-	-	
	EP + IR (SD)	-	15.4	17.3	13.3	15.1	-	-	-	
	**BLM + EP + IR (SD)**	**-**	**40.1**	**39.5**	**38.0**	**37.3**	-	-	-	
	IR (FD)	-	7.0	10.3	4.9	8.1	-	-	-	
	BLM + IR (FD)	-	7.4	11.6	5.3	9.4	-	-	-	
	EP + IR (FD)	-	8.5	10.8	6.4	8.6	-	-	-	
	**BLM + EP + IR (FD)**	-	**32.5**	**32.2**	**30.4**	**30.0**	-	-	-	
Raeisi E., 2012 [12]	CONTROLS	-	4.6		nr		-	-	-	hair loss in irradiated area
	CDDP	-	10.6		5.5		-	-	-	
	EP	-	6.6		2.0		-	-	-	
	CDDP + EP	-	20.1		15.5		-	-	-	
	IR (3 Gy)	-	15.7		11.1		-	-	-	
	CDDP + IR (3 Gy)	-	15.9		11.3		-	-	-	
	**CDDP + EP + IR (3 Gy)**	-	**30.3**		**25.7**		-	-	-	
	EP + IR (3 Gy)	-	13.4		8.8		-	-	-	
	IR (5 Gy)	-	25.2		20.6		-	-	-	
	CDDP + IR (5 Gy)	-	25.6		21.0		-	-	-	
	**CDDP + EP + IR (5 Gy)**	-	**43.2**		**38.6**		-	-	-	
	EP + IR (5 Gy)	-	22.4		17.8		-	-	-	

BLM: bleomycin; CDDP: cis-Diamminedichloroplatinum; DOX: doxorubicine; DT: tumor doubling time; EF: enhancement factor; EP: electroporation; FD: fractionated-dose irradiation; Gy: gray; IR: irradiation; OR: objective response (complete and partial responses); SD: single-dose irradiation; TCD50: radiation dose needed to control 50% of irradiated tumors; TGD: tumor growth delay; TPZ: tirapazamine. (The parts in bold highlight the association of treatments).

## Data Availability

The data presented in this study are available in this article.
